# Soluble CD26 is inversely Associated with Disease Severity in Patients with Chronic Eosinophilic Pneumonia

**Published:** 2007-02-07

**Authors:** Osamu Matsuno, Eishi Miyazaki, Shinichi Nureki, Takuya Ueno, Masaru Ando, Toshihide Kumamoto

**Affiliations:** Division of Respiratory Disease, Department of Brain and Nerve Science, Oita University Faculty of Medicine, Ufu-city, Oita 879-5593, Japan

**Keywords:** CD26, eosinophilic pneumonia

## Abstract

**Backgrounds:**

CD26, a multifunctional T cell surface glycoprotein, is a type II transmembrane protein containing only six amino acid residues in its cytoplasmic region. In addition to its membrane form, CD26 exists in plasma in a soluble form (sCD26), which is thought to be the extracellular domain of the molecule cleaved from the cell surface. Recent studies indicated CD26 have an important role in the pathogenesis of asthma, known as Th2 like disease. The function of CD26 in the esosinophlic lung disease is not well understood.

**Methods:**

Serum sCD26 was determined by enzyme-linked immunosorbent assay in patients with acute eosinophilic pneumonia, chronic eosinophilic pneumonia (CEP), and sarcoidosis, and in healthy volunteers, to establish its value for discriminating between disease entities and as marker of disease activity.

**Results:**

Soluble CD26 was significantly reduced in CEP and was related to disease severity. In particular, sCD26 was inversely correlated with arterial oxygen tension in CEP.

**Conclusion:**

Serum levels of sCD26 might appear to be useful as a new marker of CEP disease activity.

## Introduction

CD26, exhibiting dipeptidyl peptidase IV enzyme activity (DPPIV), is a multifunctional type II transmembrane glycoprotein.^1^ A soluble CD26 (sCD26), lacking the cytoplasmic tail and transmembrane region, is found in serum and other biologic fluids.

CD26 belongs to the serine protease family and cleaves N-terminal dipeptides from polypeptides with either proline or alanine residues in the penultimate position. Several cytokines and chemokines share the X-Pro or X-Ala motif at their N-terminus, including RANTES (regulated on activation normal T cell expressed and secreted), eotaxin, monocyte-derived chemokine (MDC), and stromal-derived factors (SDF-1).^2^ Thus, CD26 modulates the function of certain chemokines such as RANTES, MDC, and SDF-1.

CD26 might stimulate cellular immunity and exhibits a co-stimulatory function.^3^ CD26 has an important role in the immune system via its ability to bind adenosine deaminase^4^ and it mediates signaling by direct interaction with the cytoplasmic domain of CD45.^5^ Additionally, CD26 interacts with extracellular matrix proteins, collagen, and fibronectin.^1^

CD26 and T cells have an important role in the pathogenesis of asthma,^6^,^7^ and serum sCD26 is significantly elevated in atopic dermatitis,^8^ in which a Th2-like immune response is elicited, although the surface expression of CD26 correlates with the production of interferon (IFN)-γ in CD4+ patients with a Th1- like immune reaction. There is no information, however, about the levels of sCD26 in the serum of patients with acute eosinophilic pneumonia (AEP), chronic eosinophilic pneumonia (CEP), and sarcoidosis.

In this study, we examined the serum levels of sCD26 in patients with AEP, CEP, and sarcoidosis, and in healthy volunteers, and analyzed the possible correlation between this value and the levels of several clinical markers.

## Method

### Patients

Patient background is summarized in Table 1. The 12 patients with eosinophilic pneumonia (5 patients with AEP and 7 patients with CEP) were diagnosed at the Oita University Faculty of Medicine Hospital and related hospitals from 1999 to 2004. Pulmonary eosinophilia was detected by bronchoalveolar lavage (BAL) and transbronchial lung biopsy (TBLB) specimens. AEP was diagnosed according to the criteria described by Allen and Davis:^9^ (i) an acute febrile illness of 1–5 days duration; (ii) hypoxemic respiratory failure; (iii) diffuse alveolar or mixed alveolar and interstitial chest radiographic infiltrates; (iv) eosinophils >25% in BAL fluid; (v) the absence of parastic, fungal, or other infections; (vi) a prompt and complete response to corticoids; and (vii) the absence of relapse after discontinuation of corticoids. Most patients with AEP showed spontaneous improvement, although two patients required corticosteroid therapy for a few days. The diagnosis of CEP was based on the diagnostic criteria of CEP described by Carrington et al.^10^ CEP is characterized by fever, dyspnea, peripheral blood eosinophilia, peripheral infiltration in chest radiograph and infiltrated eosinophils in the lung. All patients were treated with corticosteroids. We also studied a control group of 27 subjects without lung disease; 13 patients with sarcoidosis and 14 healthy volunteers. Sarcoidosis was diagnosed on the basis of typical clinical features and the presence of epithelioid cell granulomas in biopsy specimens from the lung, skin, or lymph nodes. None of the patients was treated with glucocorticoids before serum sampling was completed. Informed consent was obtained from all patients and the healthy volunteers.

### Determination of serum sCD26

sCD26 concentrations in the serum were measured using a commercially available enzyme-linked immunosorbent assay kit according to the manufacturer’s protocol. A Quantikine kit from R & D Systems (Minneapolis, MN) was used to quantify sCD26. The minimal detectable level was 0.016 ng/ml. The following parameters were assessed to examine the relation between sCD26 and clinical parameters; white blood cell counts, eosinophil counts, C-reactive protein, IgE, surfactant protein D, surfactant protein A, KL-6, cell analysis in BAL, and arterial blood gas data (PaO_2_).

### Statistics

The Kruskall-Wallis test was used to compare values between groups. Where there was significant difference between groups, data were analyzed using Mann-Whitney U test. Correlation coefficients were determined by Pearson’s linear regression analysis between sCD26 and various clinical parameters. A difference was considered significant when the p-value was less than 0.05.

## Results

### sCD26 concentrations in the serum from patients with various diffuse lung diseases

**Figure 1:**
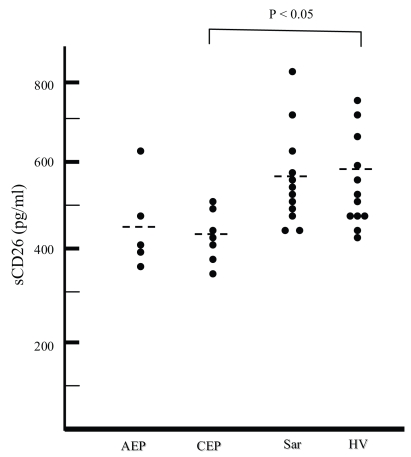
Concentration of sCD26 in serum obtained from patients with acute eosinophilic pneumonia (AEP), chronic eosinophilic pneumonia (CEP), and sarcoidosis, and healthy volunteers (HV). Significant differences are shown at the top.

sCD26 concentrations were measured by enzyme-linked immunosorbent assays. The sCD26 concentrations were significantly decreased in patients with CEP (p < 0.05), compared to healthy volunteers (Figure 1). CD26 levels in patients with AEP tended to be lower than in healthy controls, but there was no significant difference in serum sCD26 concentrations among AEP, sarcoidosis, and healthy volunteers (Figure 1). Prednisone treatment could not be responsible for the decline in sCD26, because all samples were collected before treatment.

### Relationship between sCD26 and various clinical parameters

**Figure 2:**
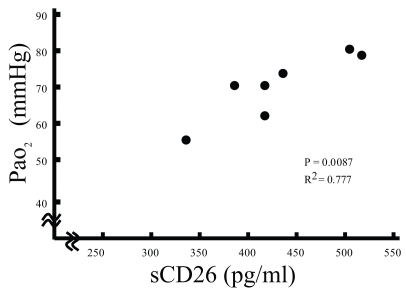
Relationship between sCD26 concentrations in the serum and PaO_2_ from patients with chronic eosinophilic pneumonia (CEP) (p = 0.0087, r^2^ = 0.777).

There was a significant correlation between PaO_2_ and sCD26 concentrations in the serum from CEP (p < 0.01; Figure 2), but not AEP (data not shown). These results suggest that serum sCD26 levels negatively related to the severity of CEP.

## Discussion

Despite the multifunctionality of CD26, the function of sCD26 in the immune response is not well understood. Upregulation of CD26 on human activated lymphocytes by interleukin (IL-12) and IL-2, but not by IL-1β, IFN-γ, tumor necrosis factor (TNF-α), or IL-4 suggests that CD26 is a marker of Th1 response.^11^,^12^ The secretion of sCD26 is not affected by IL-12, although translation and probably translocation of CD26 toward the cell surface can be regulated by IL-12.^12^ Recent studies demonstrated that tuberculous infections generate strong Th1-like response profiles. The pleural and serum CD26 levels in patients with tuberculous pleurisy were significantly higher than in patients with congestive heart failure.^13^ Immunohistochemical studies also revealed that CD26 is highly expressed in tuberculoid leprosy and sarcoidosis, known as Th1-like diseases.^14^ In our study, however, there was no significant difference in the serum levels of sCD26 between patients with sarcoidois and healthy volunteers.

The serum levels of sCD26 were significantly decreased in patients with CEP, but not AEP. CEP is a rare idiopathic inflammatory lung disease characterized by eosinophilic infiltration of the pulmonary interstitium. High levels of IL-5 have been documented in the BAL of CEP patients, consistent with this being a Th2 type inflammatory disease.^15^ CD26 and T cells have an important role in the pathogenesis of asthma.^6^,^7^ In contrast to our data, increased levels of sCD26 have been reported in patients with presumed Th2-like immune disease, such as atopic dermatitis.^8^ Recently Eltzching et al. also reported that hypoxemia induced endothelial CD26 at both the mRNA and protein.^16^ In the present study, serum levels of sCD26 were, however, inversely correlated with the disease activity of CEP patients. In another immunologic disorders, decreased serum CD26 correlates with disease severities. For example, in rheumatoid arthritis,^17^ Crohn’s disease,^18^ and systemic lupus erythematosus,^19^ CD26 levels in the serum inverse correlates with disease activity. These results indicate that CD26 has an important role not only in pathogenesis of Th1 dominant diseases, but also in Th2 dominant disease. The measurement of serum sCD26 might be of value in conjunction with other T cell activation markers to monitor both Th1-like and Th2-like immune activation. The link between reduced sCD26 levels and promotion of inflammation has been established in rheumatoid arthritis, Crohn’s disease, systemic lupus erythematosus, and CEP, but the exact role of CD26 remains obscure.

In conclusion, we demonstrated for the first time that a decrease in sCD26 is inversely associated with the severity of CEP, although our study was limited and preliminary character of findings. Serum levels of CD26 might appear to be useful as a new CEP disease activity measure.

## Figures and Tables

**Table 1:** Subject characteristics.

	Patients with eosinophilic pneumonia			
	AEP	CEP	Sarcoidosis	HV
Case (male/female)	5 (3/2)	7 (3/4)	13 (4/9)	14 (2/12)
Age (± SEM)	18.2 ± 0.53	63.5 ± 1.67	48 ± 2.53	40.3 ± 1.64

Definition of abbreviations: AEP = acute eosinophilic pneumonia; CEP = chronic eosinophilic pneumonia; Sar = sarcoidosis. Data are expressed as mean ± SEM.

## References

[1] De Meester I Korom S Van Damme J Scharpe S 1999 CD26, let it cut or cut it down Immunol Today 20 367 375 10431157 10.1016/s0167-5699(99)01486-3

[2] Mentlein R 1999 Dipeptidyl-peptidase IV (CD26)—role in the inactivation of regulatory peptides Regul Pept 85 9 24 10588446 10.1016/s0167-0115(99)00089-0

[3] Dang NH Torimoto Y Sugita K Daley JF Schow P Prado C Schlossman SF Morimoto C 1990 Cell surface modulation of CD26 by anti-1F7 monoclonal antibody. Analysis of surface expression and human T cell activation J Immunol 15 1453963 3971 1979581

[4] Dong RP Tachibana K Hegen M Munakata Y Cho D Schlossman SF Morimoto C 1997 Determination of adenosine deaminase binding domain on CD26 and its immunoregulatory effect on T cell activation J Immunol 15 1596070 6076 9550406

[5] Ishii T Ohnuma K Murakami A Takasawa N Kobayashi S Dang NH Schlossman SF Morimoto C 2001 CD26-mediated signaling for T cell activation occurs in lipid rafts through its association with CD45RO Proc Natl Acad Sci USA 9 9812138 43 10.1073/pnas.211439098 PMC59781 11593028

[6] Kruschinski C Skripuletz T Bedoui S Tschernig T Pabst R Nassenstein C Braun A von Horsten S 2005 CD26 (dipeptidyl-peptidase IV)-dependent recruitment of T cells in a rat asthma model Clin Exp Immunol 139 17 24 15606609 10.1111/j.1365-2249.2005.02666.x PMC1809259

[7] Ohnuma K Yamochi T Hosono O Morimoto C 2005 CD26 T cells in the pathogenesis of asthma Clin Exp Immunol 139 13 6 15606608 10.1111/j.1365-2249.2005.02683.x PMC1809268

[8] Katoh N Hirano S Suehiro M Ikenaga K Yamashita T Sugawara N Yasuno H 2000 Soluble CD30 is more relevant to disease activity of atopic dermatitis than soluble CD26 Clin Exp Immunol 121 187 192 10931130 10.1046/j.1365-2249.2000.01286.x PMC1905715

[9] Allen JN Pacht ER Gadek JE 1989 Acute eosinophilic pneumonia as a reversible cause of noninfectious respiratory failure N Engl J Med 321 569 74 2761601 10.1056/NEJM198908313210903

[10] Carrington CB Addington WW Goff AM 1969 Chronic eosinophilic pneumonia N Engl J Med 10 280787 98 10.1056/NEJM196904102801501 5773637

[11] Cordero OJ Salgado FJ Vinuela JE Nogueira M 1998 Interleukin-12-dependent activation of human lymphocyte subsets Immunol Lett 61 7 13 9580431 10.1016/s0165-2478(97)00154-5

[12] Salgado FJ Vela E Martin M Franco R Nogueira M Cordero OJ 2000 Mechanisms of CD26/dipeptidyl peptidase IV cytokine-dependent regulation on human activated lymphocytes Cytokine 12 1136 1141 10880264 10.1006/cyto.1999.0643

[13] Oshikawa K Sugiyama Y 2001 Elevated soluble CD26 levels in patients with tuberculous pleurisy Int J Tuberc Lung Dis 5 868 872 11573900

[14] Scheel-Toellner D Richter E Toellner KM Reiling N Wacker HH Flad HD Gerdes J 1995 CD26 expression in leprosy and other granulomatous diseases correlates with the production of interferon-gamma Lab Invest 73 685 690 7474942

[15] Taniguchi H Katoh S Kadota J Matsubara Y Fukushima K Mukae H Matsukura S Kohno S 2000 Interleukin 5 and granulocyte-macrophage colony-stimulating factor levels in bronchoalveolar lavage fluid in interstitial lung disease Eur Respir J 16 959 964 11153599 10.1183/09031936.00.16595900

[16] Eltzschig HK Faigle M Knapp S Karhausen J Ibla J Rosenberger P Odegard KC Laussen PC Thompson LF Colgan SP 2006 Endothelial catabolism of extracellular adenosine during hypoxia: the role of surface adenosine deaminase and CD26 Blood 108 1602 1610 16670267 10.1182/blood-2006-02-001016 PMC1895500

[17] Busso N Wagtmann N Herling C Chobaz-Peclat V Bischof-Delaloye A So A Grouzmann E 2005 Circulating CD26 is negatively associated with inflammation in human and experimental arthritis Am J Pathol 166 433 442 15681827 10.1016/S0002-9440(10)62266-3 PMC1602320

[18] Hildebrandt M Rose M Ruter J Salama A Monnikes H Klapp BF 2001 Dipeptidyl peptidase IV (DP IV, CD26) in patients with inflammatory bowel disease Scand J Gastroenterol 36 1067 1072 11589380 10.1080/003655201750422675

[19] Kobayashi H Hosono O Mimori T Kawasaki H Dang NH Tanaka H Morimoto C 2002 Reduction of serum soluble CD26/dipeptidyl peptidase IV enzyme activity and its correlation with disease activity in systemic lupus erythematosus J Rheumatol 29 1858 1866 12233879

